# Anti-Inflammatory Iridoids of Botanical Origin

**DOI:** 10.2174/092986712800229005

**Published:** 2012-05

**Authors:** A Viljoen, N Mncwangi, I Vermaak

**Affiliations:** Department of Pharmaceutical Sciences, Faculty of Science, Tshwane University of Technology, Private Bag X680, Pretoria 0001, South Africa

**Keywords:** Anti-inflammatory, botanical, inflammation, iridoid, natural products, NSAIDs.

## Abstract

Inflammation is a manifestation of a wide range of disorders which include; arthritis, atherosclerosis, Alzheimer’s disease, inflammatory bowel syndrome, physical injury and infection amongst many others. Common treatment modalities are usually non-steroidal anti-inflammatory drugs (NSAIDs) such as aspirin, paracetamol, indomethacin and ibuprofen as well as corticosteroids such as prednisone. These however, may be associated with a host of side effects due to non-selectivity for cyclooxygenase (COX) enzymes involved in inflammation and those with selectivity may be highly priced. Thus, there is a continuing search for safe and effective anti-inflammatory molecules from natural sources. Research has confirmed that iridoids exhibit promising anti-inflammatory activity which may be beneficial in the treatment of inflammation. Iridoids are secondary metabolites present in various plants, especially in species belonging to the Apocynaceae, Lamiaceae, Loganiaceae, Rubiaceae, Scrophulariaceae and Verbenaceae families. Many of these ethnobotanicals have an illustrious history of traditional use alluding to their use to treat inflammation. Although iridoids exhibit a wide range of pharmacological activities such as cardiovascular, hepatoprotection, hypoglycaemic, antimutagenic, antispasmodic, anti-tumour, antiviral, immunomodulation and purgative effects this review will acutely focus on their anti-inflammatory properties. The paper aims to present a summary for the most prominent iridoid-containing plants for which anti-inflammatory activity has been demonstrated *in vitro* and / or *in vivo*.

## 1 INTRODUCTION

**Fig. (1):**
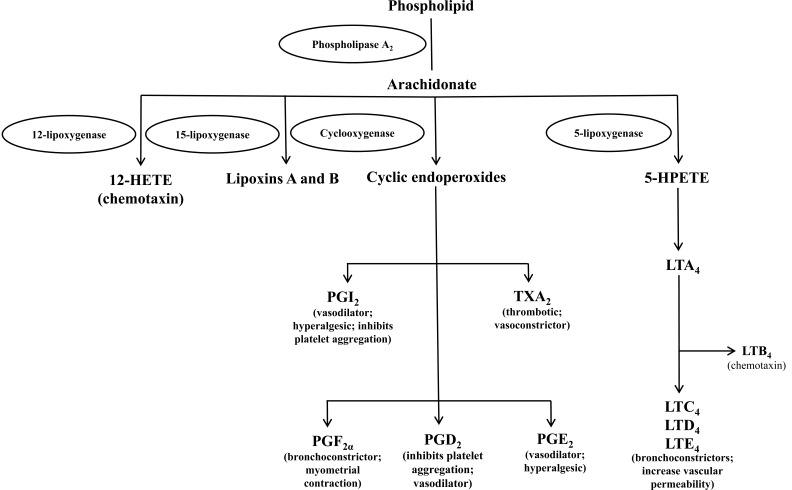
Summary diagram of mediators derived from phospholipids and their physiological effects. HETE = hydroxyeicosatetraenoic acid; HPETE = hydroperoxyeicosatetranoic acid (adapted from Rang *et al*. [3]).

Inflammation occurs as a reaction to injurious stimuli such as infection or in some cases, auto-immunity. Vasoactive amines, peptides and free radicals are some of the inflammatory mediators [1]. Inflammation is evidenced by increased temperature on site, redness, pain and swelling. It is the body’s attempt to eliminate exogenes, without which infections and wounds would not be able to heal. Macrophages, dendritic cells, histiocytes, Kupffer cells and mastocytes initiate acute inflammation after undergoing activation and release of inflammatory mediators. Vasodilation and its resulting increased blood flow cause the redness and increased heat. Increased permeability of the blood vessels results in an exudation of plasma proteins and fluid into the tissue, which manifests itself as swelling. Some of the released mediators such as bradykinin increase the sensitivity to pain. The mediator molecules also alter the blood vessels for extravasation. The neutrophils migrate along a chemotactic gradient created by the local cells to reach the site of injury and loss of function as the result of a neurological reflex in response to pain [2]. Eicosanoids are signaling molecules mainly involved in inflammation and as messengers in the central nervous system, these molecules however are not preformed in the tissues; they are generated from phospholipids. They are implicated in the control of many physiological processes and are among the most important mediators and modulators of the inflammatory reaction. The main source of eicosanoids is arachidonic acid (5, 8, 11, 14-eicosatetraenoic acid), a 20-carbon unsaturated fatty acid containing four double bonds. The principal eicosanoids are prostaglandins (PG), thromboxanes (TBX) and leukotrienes (LT); other derivatives of arachidonate such as lipoxins are also produced (Fig. **1**). The initial and rate-limiting step in eicosanoid synthesis is the liberation of arachidonate, either in a one-step or two-step process. The one step process involves phospholipase A_2_; the two-step process involves either phospholipase C or then diacylglycerol lipase or phospholipase D then phospholipase A_2_ [3]. Prostaglandins are small lipid molecules which are involved in a number of physiological processes including kidney function, platelet aggregation, neurotransmitter release and modulation of the immune system [4]. The synthesis, metabolism and signalling of the five major prostaglandins; PGD_2_, PGE_2_, PGF_2α_, PGI_2_ and thromboxane are regulated by type-specific and PG-specific pathways. COX-1 and COX-2 enzymes convert arachidonic acid liberated from membrane phospholipids to an unstable PGG_2_ intermediate which is further converted to PGH_2_ by endoperoxidase activity [5].

Almost all acute and chronic diseases are either driven or modulated by inflammation. The complexity of the interplay between beneficial and harmful effects of the inflammatory response may be the reason why there is a lack of effective therapies for many diseases [6]. Cytokines are a large group of multifunctional substances which are involved in the inflammatory response classified either as pro-inflammatory or anti-inflammatory, depending on the way they influence inflammation. Pro-inflammatory cytokines including interleukin-1β (IL-1β), tumour necrosis factor-α (TNF-α), IL-6 and IL-18 initiate and amplify the inflammatory process whereas anti-inflammatory cytokines such as IL-10, the inflammatory receptor agonist (IRA) and transforming growth factor (TGF-β) negatively modulate these events [7]. Nuclear factor kappa B (NF-κB) is a transcription factor involved at the downstream stage of many signaling cascades and plays an important role in chronic inflammation [8]. There is evidence that cytokines and their receptors are involved in the pathophysiology of many inflammatory diseases such as Alzheimer’s disease, type two diabetes mellitus, inflammatory bowel disease, sepsis, rheumatoid arthritis, atherosclerosis and asthma where there seems to be an imbalance of the cytokine network and excessive recruitment of leukocytes to inflammatory sites [7;9]. Anti-cytokines which still require further research may be beneficial in cystic fibrosis where there is persistent and dysregulated inflammation, combined with exaggerated immune response [10]. There is evidence from preclinical and clinical studies that persistent inflammation is a driving force in the development of carcinogenesis. Various mechanisms are involved in this process; these include the induction of genomic instability, alterations in epigenetic events and subsequent inappropriate gene expression, enhanced proliferation of initiated cells, resistance to apoptosis, aggressive tumour neovascularisation, invasion through tumour-associated basement membrane and metastatis [11]. Inflammation has also been implicated in coronary diseases such as atherogenesis, atherosclerotic plaque progression and acute coronary syndrome. C-reactive protein is used as a common marker for disease progression [12].

The common signs of arthritis such as redness, swelling and pain occurs as a result of inflammation. This disease affects about 1% of the US adult population, with rheumatoid arthritis being the most common form. It is three times more common in women than in men [13]. The joints of the knees, hips and wrists are the common sites of the disease; however other joints may also be affected. Patients suffering from rheumatoid arthritis often present with ocular and pulmonary inflammation, nodules on the extensor surfaces of the elbows, lymphadenopathy and splenomegaly additional to the classical inflammation of the joints [13]. The disease is likely to go undiagnosed for many years, firstly because of its *‘wear and tear’* nature which is regarded as the normal process of ageing and also because of the availability of drugs used for the treatment of inflammation which is a classic sign and symptom of rheumatoid arthritis. The exact pathophysiology of the disease is poorly understood; and to understand it better, it is commonly classified according to where the inflammation occurs in the body, for example, joints, spine and soft tissue rheumatoid arthritis [14]. It is thought that rheumatoid arthritis results from a dysfunctioning of the immune system. Rheumatoid arthritis patients have been shown to have elevated levels of IL-1 and TNF-α [15] and activation-induced, T-cell derived, chemokine-related cytokine/lymphotactin [16] as well as macrophage migration inhibitory factor [17]. These endogenous compounds stimulate synovial tissue effector functions, including proliferation, matrix metalloproteinases (MMPs) expression, adhesion-molecular expression, secretion of other cytokines and prostaglandin production, all of which may play a role in the pathogenesis of rheumatoid arthritis [18]. 

However, genetic factors have also been indicated to have an influence on the pathogenesis of the disease [19-20]. Studies have demonstrated that most patients express specific human lymphocyte antigens in the major histocompatability complex located on T-lymphocytes. Both humoral and cellular immunologic mechanisms are involved in the pathogenesis of the disease. The mechanisms include cytokine-mediated activation of T and B lymphocytes and the recruitment and activation of polymorphonuclear leukocytes. The inflammatory leukocytes then release a variety of prostaglandins, cytotoxic compounds and free radicals that cause joint inflammation and destruction [21]. Acute inflammation is necessary for eliminating exogenes and promoting coagulation, however, left unchecked this process may trigger carcinogenesis, which increases cell differentiation and cell up-regulation which leads to abnormal tissue growth, and ultimately cancer [22]. 

## 2 ALLOPATHIC MEDICINES COMMERCIALLY AVAILABLE USED TO TREAT INFLAMMATORY DISEASES 

Inflammation may be involved in the pathophysiology of a host of diseases; however, rheumatoid arthritis remains an epitome of inflammatory diseases. It is a progressive disease and lifestyle plays an important role in disease progression and even prognosis. Patients suffering from this disease have been shown to be amongst the ones who are more likely to self-diagnose and self-prescribe. Non-steroidal anti-inflammatory drugs (NSAIDs) are the first-line treatment for inflammatory disorders, with the classic example being ibuprofen. These are widely available over the counter, without a prescription and are prone to irrational prescribing and abuse due to the epidemiology of the disease and its impediment on the quality of life of these patients. However, this group of drugs remains the mainstay as potent anti-inflammatory and analgesic agents. Non-steroidal anti-inflammatory drugs are weakly acidic drugs which exert their action by inhibiting the COX enzyme. This forms the basis of their classification; selective and non-selective COX inhibitors. COX is an enzyme that catalyses the conversion of arachidonic acid into arachidonate from which the prostaglandins are synthesised. Nonsteroidal anti-inflammatory drugs also inhibit B and T cell proliferation by mechanisms that do not involve inhibition of COX and prostaglandin formation. Prostaglandins are necessary for the maintenance of the gastric mucosa and its protection from the acidic gastric fluids. Thus, inhibition of the COX-1 enzyme which is directly involved in this function results in gastric erosion which ultimately leads to gastric ulceration and/or perforation. COX-2 is the isoform which is responsible for inflammation orchestration [23]. 

The coxibs, a newer class of selective COX-2 inhibitors, have gained favour for the treatment of rheumatoid arthritis although at a significant price increment [24]. Pharmaco-economical studies have revealed that the risk/benefit ratio justifies the price and in addition improves the quality of life of patients. Additionally, these drugs have a prolonged half-life of approximately 12 h which allows for a twice daily dosing, not only for better patient compliance, but it also offers the much needed relief from the pain and loss of joint function associated with rheumatoid arthritis for an extended period of time. Although they offer several health benefits; selective COX-2 inhibitors have been shown to be associated with cardiovascular side effects. Using mouse models, Narasimba and co-workers demonstrated that COX-2 deficiency contributes to the pro-atherogenic properties of high density lipoproteins [25].

Rheumatoid arthritis is a chronic disease, thus there is a continuous search for remedies which are safer for long-term use. Non-steroidal anti-inflammatory drugs are generally well-tolerated. When taken on a short-term basis, dyspepsia and stomach discomfort are common but tolerable side effects [26]. However, on long-term basis, gastrointestinal erosion evidenced by gastric ulceration and perforation has been recorded and may even be lethal [27-28]. Non-steroidal anti-inflammatory drugs are extensively metabolised in the liver and excreted in the urine and faeces. Liver and kidney failure has been reported in patients who failed to adequately hydrate themselves during NSAID therapy [26; 29]. Groups at risk are the elderly and young children because of reduced hepatic and renal function. Additionally, in young children there is misuse of NSAIDs, where misinformed mothers use NSAIDs for their anti-pyretic properties whereas there are safer alternatives, but the side effects ensue nonetheless. Omeprazole, a substituted benzimidazole is a potent inhibitor of gastric acid secretion which interacts with the gastric proton pump (K^+^/H^+^ -ATPase) in the parietal cell secretory membrane [30]. This drug is often prescribed for chronic ulceration commonly associated with NSAID treatment. Researchers have however reported that omeprazole and its metabolites may decrease natural killer cell cytotoxic activity [31]. 

Corticosteroids and their biologically active synthetic derivatives are employed when endogenous production is impaired, which may be the case in some inflammatory and auto-immune diseases such as inflammatory bowel syndrome, asthma and arthritis. Glucocorticoids are potent immuno-suppressors and anti-inflammatory agents; and are thus frequently prescribed for inflammatory and auto-immune diseases [2]. Use of corticosteroids requires close monitoring because their side-effects are widespread to every organ of the body. Side effects are dose-related, they include, fat redistribution, rounded plethoric face and purple striae. Their mechanism of action involves binding to the cytoplasmic glucocorticoid receptor; the activated receptor-glucocorticoid complex enters the cell nucleus and binds to steroid response elements on target DNA molecules. This induces synthesis of specific mRNA or represses genes by inhibiting transcription factors such as NFκB. Synthetic glucocorticoids have higher affinity for this receptor [32]. 

## 3 IRIDOID CHEMISTRY AND RESEARCHED ANTI-INFLAMMATORY IRIDOID-CONTAINING PLANTS

Nature provides a wide range of compounds with a similarly wide range of biological activities. One such class of compounds is the iridoids. These metabolites are present in plants and animals; in plants they are often bound to glucose thus referred to as iridoid glycosides. Iridoids represent a large group of cyclopenta[c]pyran monoterpenoids that are abundant in Dicotyledonous plant families and especially in sympetalous families such as the Apocynaceae, Scrophulariaceae, Verbenaceae, Lamiaceae, Loganiaceae and Rubiaceae etc. [33]*. In vitro* and *in vivo* studies have revealed that iridoids have neuroprotective, anti-inflammatory, immuno-modulating, hepatoprotective, cardioprotective, anticancer, anti-oxidant, antimicrobial, hypoglycaemic, hypolipidaemic, choleretic, antispasmodic and purgative properties [34-40]. In terms of chemical structure, iridoids are characterised by a six-membered ring containing an oxygen bound to a cyclopentane ring. 


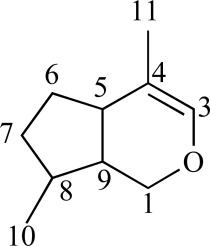


The iridane skeleton is formed by the cyclisation of 10-oxogeranial biosynthesised from geraniol through 10-hydroxygeraniol to yield iridodial, which is subsequently oxidised to iridotrial. Glycosylation, methylation and oxidation etc. converts iridotrial to iridoid compounds. Various classifications have been proposed, but most seem to agree that there are four major iridoid groups. Iridoid glycosides have glycosidic linkages, usually formed at the aglycone C-1 or C-11 hydroxyl. Non-glycosylated or simple iridoids (lognic acid; loganin) have an iridane skeleton with a methyl at C-8 and another carbon bonded to C-4. Secoiridoids, such as gentiopicroside and sweroside, are formed through cleavage of the 7,8-bond of the cyclopentane ring. Almost all secoiridoids are glycosides. Dimerisation of iridoids and secoiridoids lead to the formation of bisiridoids [33; 41-42].

Several structure-activity relationships were deduced in a study by Recio *et al.* [43]. If a hydroxyl function is introduced, the topical activity decreases. Hence harpagoside and lamiide are less effective topically than loganin. The conversion of a –COOH moiety to its –COOMe analogue increases topical activity. Other positive characteristics for topical activity are hydroxyl substitution at C-5, unsaturation at C7-C8 and methyl substitution of carboxyl C-11. The most positive characteristic for anti-inflammatory activity is a double bond between C-7 and C-8 [43]. Studies have shown that the activity of iridoid glycosides increase after hydrolysis. Both harpagide and harpagoside exhibited no inhibition of COX-1 and COX-2 enzymes, TNF-α release or NO production. After hydrolysis with β-glucosidase, harpagide is transformed into H-harpagide while harpagoside is transformed to H-harpagide and cinnamic acid. The hydrolysed product, H-harpagide significantly inhibited COX-2 activity and it was noted that the chemical structure of this compound was similar to PGE_2_ and other COX-2 selective inhibitors such as celecoxib. These compounds have a pentanomic ring with two adjacent side-chains and the similarity may be responsible for the binding to the COX-2 enzyme [44]. Park *et al. *[45] hydrolysed several iridoid glycosides (catalpol, gentiopicroside, loganin etc.) using β-glucosidase to show their inhibitory effects. Species from which iridoid glycosides have been isolated and tested are discussed hereafter; Table **1** summarises species; compound(s) isolated; mechanism of action and whether *in vivo* or *in vitro* data has been generated. 

The species included in this review have been selected after several searches were completed. Both Scopus and SciFinder was consulted using various search terms such as “iridoid” AND “inflamm*”. SciFinder was used to further explore anti-inflammatory activity for iridoids using the compound name e.g. “harpagoside” AND “inflamm” as search terms. The bibliographies of all relevant papers were further consulted to obtain any relevant papers. The premise on which botanical species were selected for discussion was; 1. if an extract of that particular species had been assayed to determine anti-inflammatory properties (*in vitro* and *in vivo*) and 2. if iridoids were shown to possibly contribute to the observed activity. Screening-type papers where no information was provided on the chemical composition / profile of the extracts were not considered. Clearly, a specific iridoid may not be restricted to a single species but may occur in related or taxonomically distant taxa. Although it may be reasonable to extrapolate research from one study to another species containing the same iridoid, it was not the purpose of this review to provide an exhaustive compilation of all iridoid-containing species and to speculate on their possible anti-inflammatory activity. 

### 3.1 *Ajuga bracteosa* (Bugleweed)

*Ajuga bracteosa* Wall. ex Benth. (Lamiaceae) is a perennial herb that grows in India and Taiwan where it is traditionally used to treat inflammatory disorders such as hepatitis, pneumonia and bone disease. In India it is a known remedy for malaria and in Ayuverda for the treatment of rheumatism, gout, palsy and amenorrhoea. This plant is also renowned for its astringent, febrifugal, stimulant and diuretic properties [46-48]. *Ajuga bracteosa* contains sphingolides, bractic acid, diterpenoids and withanolides which inhibit enzymes such as lipoxygenase (LOX) and acetylcholinesterase (AChE) [47]. Scientific studies revealed chemopreventitive, antiplasmodial and cardiostimulant effects [46; 48]. 

Gautam *et al. *[46] evaluated the topical anti-inflammatory effect of a 70% ethanol extract of the whole plant in 12-*O*-tetradeconoylphorbol-13-acetate (TPA)-induced ear oedema in female Swiss albino mice. The extract showed significant dose-dependent anti-inflammatory activity at doses of 0.5 and 1.0 mg/ear as measured by ear thickness. Testing with an EIA kit showed strong inhibitory activity of 50.56±1.12% (COX-1) and 42.38±0.90% (COX-2) at 25 μg/mL, and 79.33±1.09% (COX-1) and 68.80±0.54% (COX-2) at 50 μg/mL. The COX-1 and COX-2 inhibitory activity of five compounds, ajugarin I, 6-deoxyharpagide (**1**), lupulin A, reptoside (**2**) and withaferin A, isolated from this extract were determined at a concentration of 30 μM. The two iridoid glycosides (reptoside and 6-deoxyharpagide) exhibited mild to moderate inhibitory activity of 33.55±0.76% (COX-1) and 51.30±1.56% (COX-2) for reptoside and 38.36±2.01% (COX-1) and 59.45±0.66% (COX-2) for 6-deoxyharpagide. Of the five isolated compounds, 6-deoxyharpagide exhibited the highest COX-2 activity. The activity of the iridoid glycosides reptoside and 6-deoxyharpagide as well as clerodane diterpenes and withaferin A may thus be responsible in part for the anti-inflammatory activity of the extract [46]. A 70% ethanolic extract produced anti-arthritic effects in Wistar albino rats after joint oedema was induced. The extract protected against swelling produced by administration of turpentine oil and significant inhibition of joint oedema (81.08%; 20 mg/kg) after formaldehyde administration was seen compared to aspirin (40.51%; 100 mg/kg). The *A. bracteosa* extract showed promising anti-arthritic activity against both acute and chronic arthritis which supports its traditional use [48].

### 3.2 *Boschniakia rossica* (Northern groundcone)

*Boschniakia rossica* (Cham. & Schltdl.) Standl. (Orobanchaceae) is a parasitic plant growing on the roots of *Alnus* species (Betulaceae). It is a most valuable medicinal plant in China and is also found in the Democratic People’s Republic of Korea, Japan and Russia. *Boschniakia rossica* is widely used in Chinese traditional medicine as a substitute for *Cistanchis Herba*, a famous stamina tonic agent and it is a well-known anti-senile agent consumed as an alcoholic infusion [49-50]. Several compounds have been isolated from *B. rossica *including phenylpropranoid and iridoid glycosides [51]. These compounds have been shown to exhibit anti-inflammatory, anti-lipid, peroxidative and free-radical scavenging activities [52]. Quan *et al.* [50] reported that a *B. rossica n*-butanol extract consisted of the iridoid glycosides boschnaloside (30.1%) (**3**) and 8-epideoxyloganic acid (16.6%) (**4**), as well as the phenylpropanoid glycoside rossicaside B (32.2%). Administration of the extract to rats resulted in the suppression of TNF-α, iNOS and COX-2 protein secretion and/or enhanced the degradation of these proteins [50]. The anti-inflammatory activity of a water and dichloromethane extract fractionated from a methanol extract was tested after xylene-induced ear swelling in mice (Kunming strain). The water (8.9-28.8%) and dichloromethane (26.2-27.4%) extracts as well as the control, indomethacin (18.8-21.5%), inhibited the degree of swelling significantly compared to the normal saline group [53]. The same authors investigated the effect of the extracts in rats and mice. The water extract exhibited inhibitory effects on acute inflammation in the carrageenan-induced paw oedema, hot scald-induced paw oedema, and histamine-induced oedema assays as well as on chronic inflammation in the adjuvant-induced arthritis and cotton pellet-induced granuloma formation assays [49]. 

Lin *et al.* [51] evaluated the anti-inflammatory activity of boschnaloside (**3**) and 8-epideoxyloganic acid (**4**) through its effects on *N*-formyl-methionyl-leucyl-phenylalanine (fMLP) and phorbol-12-myristate-13-acetate-(PMA)-activated peripheral human neutrophils (PMNs) and mononuclear cells. These iridoid glycosides exhibited activity with an IC_50 _value of 8.9-28.4 μM in PMA-activated PMNs and 19.1-21.1 μM in fMLP-activated mononuclear cells. This indicated the potential of iridoid glycosides to affect the inflammatory process through the inhibition of ROS production and/or providing a radical scavenging effect during oxidative stress [51]. Boschnaloside has been isolated from several other species including *Euphasia pectinata *[54] and* Pedicularis verticillata *[55]. 

### 3.3 *Bouchea fluminensis* (Wandering Jew)

The genus *Bouchea* has a limited geographical distribution in the Western hemisphere with only one out of ten species, *Bouchea pterygocarya*, occurring in the Eastern hemisphere. *Bouchea fluminensis* (Vell.) Moldenke, a member of the Verbenaceae family, is a herbaceous plant found in Brazil and Bolivia. Traditionally, an infusion of its aerial parts is widely used for its bowel-stimulating and regulating properties on digestive functions and as an anti-inflammatory agent. Together with species of *Stachytarpheta*, it is used in folk medicine in Brazil to treat gastric disorders and as an anti-emetic [56-57]. Chemical analyses performed on extracts of this plant have identified lamiide (**5**) as the main component [58].

Delaporte *et al.* [56] assessed lamiide (**5**) for anti-inflammatory activity in the carrageenan-induced rat-paw oedema and rat-brain phospholipid assays. Lamiide (12.5-100 mg/kg) was orally administered to female Wistar rats 30 min prior to carrageenan injection with indomethacin (10 mg/kg) as a positive control. Oedema increased progressively, reaching a maximum at 4 h where the volume of the carrageenan-injected paw was 42±1% greater than the saline-injected paw. The administration of lamiide reduced the oedema dose-dependently with an ED_50_ value of 62.3±7 mg/kg of weight. The increase in paw volume at 4 h was 42±1% for the saline control, 15±3% in the indomethacin group (positive control) and 17±3 and 9±1% in the 50 mg/kg and 100 mg/kg lamiide groups, respectively [56]. 

In the rat-brain phospholipid assay, the ability of lamiide to inhibit peroxidation of membrane lipids was tested. Phospholipids spontaneously undergo non-enzymatic oxidation when incubated at 37 °C in the presence of Fe^+2^. Lamiide exhibited inhibition of phospholipid peroxidation at an IC_50_ value of 0.929± 0.01 mM. The percentage inhibition was significantly different compared to the positive control, Trolox^®^ C (45%; 100 μM), with 57% and 71% inhibition at 1.20 mM and 1.40 mM, respectively. The presence of lamiide therefore may be partially responsible for its anti-inflammatory effect through its free radical scavenging activity [56]. Lamiide has also been identified in other *Phlomis* species such as *P. sintenisii *[59], *P. grandiflora *var* fimbrilligera *[60],* P. pungens* var*. pungens *[61],* P. samia *[62] and* P. aurea *[63].

### 3.4 *Catalpa ovata* (Yellow catalpa)

The stem bark of *Catalpa ovata *G. Don. (Bignoniaceae), cultivated as an ornamental tree, has been used traditionally in Korea to treat several inflammatory diseases [64]. Pae *et al.* [64] investigated the effects of the methanol extract of this folk medicine on the production of two major macrophage-derived inflammatory mediators, TNF-α and NO. The RAW 264.7 macrophages were pre-incubated with the extract and then activated with the endotoxin lipopolysaccharide (LPS). The extract dose-dependently inhibited the secretion of TNF-α and NO synthesis with a significant decrease in mRNA-expression of iNOS and intracellular TNF-α synthesis. It was also found that the extract reversed toxicity in RAW 264.7 macrophages induced by LPS and the extract itself did not show any cytotoxicity [64]. 

Catalposide (**6**), an iridoid isolated from *C. ovata*, has been investigated for its anti-inflammatory effects. Catalposide has been reported to inhibit the production of TNF-α, IL-1β and IL-6, as well as the activation of NF-κB in LPS-activated RAW 264.7 macrophages. In addition, it inhibited the expression of these genes and the nuclear translocation of the p65 subunit of NF-κB. A possible mechanism of action was deduced in that catalposide inhibited the binding of LPS to RAW 264.7 cells [65]. Another study revealed that catalposide significantly inhibited the production of NO in a dose-dependent manner in LPS-stimulated RAW 264.7 macrophages, suppressed the expression of the iNOS gene and protein and inhibited the activation of LPS-induced NF-κB [66]. In human intestinal epithelial HT-29 cells, catalposide attenuated the TNF-α-dependent expression of the pro-inflammatory gene IL-8. The TNF-α-induced IL-8 secretion was reduced in a dose-dependent manner with optimal inhibition at ≥ 200 ng/ml. An *in vivo* study was done to determine whether catalposide could affect intestinal inflammation using the trinitorbenzene sulfonic acid (TNBS)-induced inflammatory colitis mouse model. TNBS was administered intrarectally in 2 doses (7 days apart) to induce colitis, causing severe bloody diarrhoea, rectal prolapse and wasting. Catalposide (10 μg/mouse) was administered *via *the lumen of the colon 1 day before and 3 and 6 days after the first TNBS administration. Treated mice showed striking improvement of the wasting disease through a fast and dramatic increase in bodyweight. Macroscopic analysis of the colon revealed that catalposide prevented both hyperaemia and inflammation and histopathologic analysis showed that catalposide restored the histologic appearance of the mucosa and submucosa. In addition, it was shown that catalposide inhibited NF-κΒ *in vivo* in addition to its *in vitro* action. Therefore, oral administration of catalposide may be helpful in the treatment of inflammatory bowel disease (IBD) in humans [67], and clinical trials to substantiate its traditional use should be conducted. 

### 3.5 *Cornus officinalis* (Japanese cornel; Dogwood fruit)

*Cornus officinalis *Siebold et Zucc is a member of the Cornaceae family. Use of this herb was first recorded in Shen Nong’s *Materia* *Medica *about 2000 years ago in China. Cornel iridoid glycoside (CIG) is a main component extracted from *C. officinalis*. Additionally, the extract of *C. officinalis* is composed of organic acids, polysaccharides, saponins and iridoids such as oleanolic acid, ursolic acid, morroniside (**7**), loganin (**8**), sweroside (**9**), and cornuside (**10**) [68-69]. *Cornus officinalis* is a traditional Oriental medicine credited with curing inflammatory diseases and invigorating blood circulation. Several biological activities are ascribed to *C. officinalis* including hepatic function improvement, antimicrobial activity, antineoplastic, anti-inflammatory and antidiabetic effects [70-71]. 

Yao *et al.* [68] investigated the effects of cornel iridoid glycoside (CIG), which is a combination of morroniside (**7**) and loganin (**8**), on neurological function and neurogenesis after ischaemic stroke. CIG was intragastrically administered to rats in doses of 20, 60 and 180 mg/kg/day, starting 3 h after the onset of middle cerebral artery occlusion. The treatment with CIG at the doses of 60 and 180 mg/kg/day significantly improved neurological function, and increased the number of bromodeoxyuridine-positive cells and nestin-positive cells in the subventricular zone of rats 7, 14 and 28 days after ischaemia. The results indicated that CIG promoted neurogenesis and angiogenesis and improved neurological function after ischaemia in rats [68]. This shows that *C. officinalis* may be beneficial in conditions such as atherosclerosis which may be a predisposing factor for an ischaemic stroke. The anti-inflammatory effect of *Cornus officinalis *glycosides (COG) was investigated in rats using the Freund’s adjuvant-induced arthritis method. Significant suppression of oedema was noted as well as inhibition of IL-1, IL-6 and TNF-α in peritoneal macrophages as well as prostaglandin E2 (PGE_2_) in plasma [72]. An aqueous extract prepared from the fruit inhibited LPS-induced expression of COX-2 and iNOS in RAW 264.7 macrophages. In addition, it suppressed PGE_2_ synthesis, NO production and NF-κB levels in the nucleus. In addition, the acetic acid-induced writhing response in mice was suppressed indicating analgesic action [73].

Yamabe *et al.* [74] showed that loganin (**8**) exhibits protective effects against hepatic injury and other diabetic complications associated with abnormal metabolic states and inflammation caused by oxidative stress and advanced glycation end-product formation. In a recent study, a morroniside cinnamic acid conjugate was prepared and evaluated on E-selectin mediated cell–cell adhesion as an important role in inflammatory processes. 7-*O* Cinnamoylmorroniside (**11**) exhibited anti-inflammatory activity (IC_50_ = 49.3 µM) by inhibiting the expression of E-selectin and was observed to be a potent inhibitor of TNF-α-induced E-selectin expression [75]. It was also determined that morroniside isolated from the fruits of *C. officinalis* inhibited the formation of reactive oxygen species (ROS) and lipid peroxidation and down-regulated the expression of NF-κBp65, COX-2 and iNOS which is increased in type-2 diabetic mice [69]. 

Cornuside (**10**), a bisiridoid glycoside compound isolated from the fruit, suppressed cytokine-induced pro-inflammatory and adhesion molecules in human umbilical vein endothelial cells (HUVECs). Cornuside attenuated TNF-α-induced NF-κBp65 translocation, suppressed the expression of intercellular adhesion molecule-1 (ICAM-1), vascular cell adhesion molecule-1 (VCAM-1) and monocyte chemoattractant protein-1 (MCP-1). Therefore, it has an effect on vascular inflammation caused by an increase in leukocyte-endothelium adhesion *via *up-regulation of endothelial cell adhesion molecules (ICAM-1; VCAM-1) and pro-inflammatory factors like MCP-1, which are induced by NF-κB [70]. In another *in vitro* study it was determined that cornuside (30 μM) significantly inhibited LPS-induced production of NO (67.6%), PGE_2_, TNF-α (50.8%), IL-6 (75.7%) and IL-1β (55.4%), reduced mRNA expression of COX-2 and iNOS and attenuated the translocation of NF-κBp65. Cornuside has the potential to be a good candidate to treat inflammatory disorders if these effects could be confirmed in an *in vivo* model [76]. 

### 3.6 *Enicostema axillare* (Indian whitehead)

The perennial herb, *Enicostema axillare* (Lam.) A.Raynal (Gentianaceae) is found throughout India. Traditionally, the plant is used in folk medicine to treat diabetes mellitus, rheumatism, abdominal ulcers, hernia, swelling and itching in addition to being used as an anti-inflammatory, digestive, thermogenic and liver tonic. Anti-inflammatory, anti-oxidant, hypoglycaemic and anticancer activities of this species have been reported [77]. Compounds isolated from this species include swertiamarin (**12**), alkaloids, steroids, triterpenoids, saponins, flavonoids, xanthones and phenolic acids. Many of these compounds exhibit anti-inflammatory and anti-oxidant properties [78]. Vaijanathappa and Badami [79] investigated the antioedematogenic and free radical scavenging activity of swertiamarin (**12**) isolated from an ethyl acetate extract of *E. axillare*. In the carrageenan-induced rat-paw oedema test the results showed oedema inhibition 5 h after induction of 38.60% (swertiamarin, 100 mg/kg body weight), 52.50% (swertiamarin, 200 mg/kg body weight) and 45.55% (diclofenac sodium, 100 mg/kg/body weight). This indicated superior activity of swertiamarin at a dose of 200 mg/kg compared to the standard, diclofenac sodium [79]. The hepatoprotective and anti-oxidant activity of swertiamarin (100 and 200 mg/kg body weight) was determined after inducing liver injury in rats by administering d-galactosamine (200 mg/kg body weight) intraperitoneally. Swertiamarin (100 and 200 mg/kg body weight) was administered orally for 8 days prior to d-galactosamine. A significant normalisation of all the altered biochemical parameters was noted indicating the anti-oxidant and hepatoprotective nature of swertiamarin. Park *et al.* [45] also showed that hydrolysed swertiamarin inhibits thromboxane-B_2_ (TBX_2_). 

The *in vivo* antinociceptive activity of swertiamarin (**12**) isolated from *E. axillare* was investigated using male adult albino mice. In the hot plate method, a significant increase in the response time was observed for the 100 mg/kg body weight swertiamarin-treated group after 30 and 45 min and after 15, 30 and 45 min for the 200 mg/kg bodyweight group. The paracetamol-treated group (100 mg/kg bodyweight) showed an increase in the latency period only after 30 and 45 min. The percent protection observed after 45 min was 109.42% for the paracetamol group, 147.42% for the swertiamarin 100 mg/kg body weight group and 157.14% for the swertiamarin 200 mg/kg body weight group. A significant increase in the tail withdrawal reflex was observed for the swertiamarin-treated group with percent protections of 150% (100 mg/kg bodyweight) and 200% (200 mg/kg bodyweight), which was higher compared to paracetamol at 138% (100 mg/kg bodyweight). The intraperitoneal injection of acetic acid (0.3%, 10 ml/kg bodyweight) into control mice produced 17.83±1.10 writhes. Swertiamarin administered orally 30 min prior to the administration of acetic acid reduced the number of writhes to 8.66±0.21 (100 mg/kg bodyweight) and 7.83±0.60 (200 mg/kg bodyweight) which translates into 51.43 and 56.08% protection, respectively. Paracetamol (100 mg/kg bodyweight) reduced the number of writhes to 7.00±0.36 (60.74% protection). The dose of 200 mg/kg bodyweight of swertiamarin was found to be more potent. An acute toxicity study revealed no clinical signs of toxicity or mortality in doses of up to 2000 mg/kg bodyweight administered orally suggesting that it is safe for clinical use. These results suggest that swertiamarin possess both peripheral (acetic acid-induced writhing test) and central (hot plate and tail immersion tests) antinociceptive activity. Swertiamarin may possess central antinociceptive activity involving opioid-like receptor mediation as well as peripheral antinociceptive activity by inhibiting the synthesis or actions of prostaglandins. Further research is required to determine its mechanism of action [77]. Swertiamarin (**12**) is a common secoiridoid present in several *Swertia* and *Gentiana* species. 

### 3.7 *Eucommia ulmoides* (Hardy rubber tree)

*Eucommia ulmoides* Oliv. belongs to the Eucommiaceae family and is a relic plant that survived in China from the quaternary glacial period. Later on, it was introduced into other parts of the world, such as Japan, Korea and America. In the 1980s, uncontrolled harvesting for the bark resulted in near extinction from its natural habitat. Currently, almost all the forests of *E. ulmoides* are commercial plantations. Historically, only the bark (cortex Eucommiae) was officially recognised as a traditional Chinese tonic drug [80]. However, modern scientific research has confirmed that chemical constituents in the leaf of *E. ulmoides* are similar to those in the bark and the biological activity is also comparable. The main secondary metabolites in this plant are iridoids (e.g. geniposide (**13**) and aucubin (**14**)), phenylpropanoids (chlorogenic acid and caffeic acid) and flavonoids. Research has confirmed that these secondary metabolites demonstrate profound pharmacological activities such as lowering blood pressure and improving diabetes mellitus and anti-oxidant and antimutation activity. *Eucommia ulmoides* can also improve general health, strengthen the body, promote metabolism and rejuvenate the body [80].

A bark water extract suppressed the COX-2 enzyme at an IC_50 _value of 9.92 µg/ml but showed no effect on TNF-α and NO production in RAW 264.7 macrophages, nor on NF-κB. HPLC analysis showed the presence of catalpol (**15**) and geniposide (**13**) [81]. Liu *et al.* [82] showed that geniposide effectively inhibited LPS-induced expression of IL-6 and IL-8 in HUVECs at the transcription and translation levels. Additionally, geniposide (**13**) suppressed LPS-induced HUVEC migration and monocyte adhesion to HUVECs. Geniposide thus inhibits LPS-induced IL-6 and IL-8 production in HUVECs by blocking signaling pathways [82]. 

Aucubin (**14**) is an iridoid glycoside with a variety of pharmacological effects, such as antimicrobial and anti-inflammatory activity, whilst also promoting dermal wound healing. Shim *et al.* [83] examined the effects of 0.1% aucubin on oral wound healing. ICR male mice were divided into two groups: an untreated control group (n=18) and an aucubin-treated group (n=18). Saline or 0.1% aucubin solution was injected and artificial full thickness wounds were made on either side of the buccal mucosa. Re-epithelisation and matrix formation of the aucubin-treated group occurred earlier than that of the control group and the number of inflammatory cells of the aucubin-treated group was fewer than that of the control group.

### 3.8 *Gardenia jasminoides* (Cape Jasmine)

The dried ripe fruit of *Gardenia jasminoides* J.Ellis (Rubiaceae) is widely used in traditional medicine for its cholagogue, sedative, diuretic, antiphlogistic, anti-inflammatory, antioedematogenic and antipyretic effects in Korea, Japan and China. Scientific studies revealed anti-inflammatory, fibrolytic, anti-oxidant, anti-thrombotic, neuritogenic and cytotoxic activities [84-85]. In a recent study, geniposide (**13**), one of the main compounds of *G. jasminoides *was tested for its effectivity in the treatment of ankle sprain induced in rats. An ankle sprain was inflicted under anaesthesia and the swelling was treated topically (200 μl/24 h) starting from the 12^th^ hour after induction with an ethanolic extract of the fruit (100 mg/ml), geniposide (10 mg/ml; 100 mg/ml) and diclofenac gel (12.5 mg/ml) as control group. All the groups showed significant reduction in swelling compared to the vehicle-treated control group: ethanolic fruit extract ➔13-18%; 10 mg/ml geniposide ➔ 20-23%; 100 mg/ml genipose ➔ 21-34%; diclofenac gel 12.5 mg/ml ➔ 20-38%. These results indicate the potential beneficial effect of the ethanolic fruit extract and geniposide for the treatment soft tissue injuries [85]. 

Geniposide (**13**) also showed significant inhibition of IL-2 secretion by phorbol myristate acetate and anti-CD_28_ monoclonal antibody co-stimulated activation of human peripheral blood T cells thus proving to be immunosuppressive [86]. *Gardenia jasminoides* ethanol extract and its constituents are reported to reduce the risks of gastritis and reverse gastric lesions in rats [87]. In the carrageenan-induced rat-paw oedema assay, a fruit extract inhibited oedema at doses of 50 mg/kg (10.2%), 100 mg/kg (25.9%), 200 mg/kg (28.6%) and 400 mg/kg (35.8%). Geniposide (100 mg/kg) inhibited oedema by 31.7% measured 3 h after carrageenan injection. The extract also inhibited vascular permeability by possibly protecting against the release of inflammatory mediators and dose-dependently inhibited acetic acid-induced abdominal writhing in mice (80.1% at 200 mg/kg). This indicated analgesic activity in addition to anti-inflammatory activity. Geniposide (**13**) also inhibited the production of carrageenan-induced formation of exudates and nitric oxide [84]. The effect of geniposide acid on adjuvant-induced arthritis was investigated using male Wistar rats. Paw swelling was significantly reduced and the level of TNF-α and IL-1β in rat serum decreased [88].

Geniposide (**13**) is transformed in body tissues to its aglycone, genipin (**16**). Geniposide (**13**) is first hydrolysed to genipin (**16**) by β-glucosidases and subsequently to the aglycone of geniposidic acid (**17**) by esterases. Thus, when geniposide is orally administered, genipin is produced in the intestine [89]. In RAW 264.7 cells, functional significance of heme oxygenase-1 (HO-1) induction was revealed by genipin-mediated inhibition of LPS-stimulated iNOS expression or COX-2 promoter activity. The response was reversed by the blocking of HO-1 protein synthesis or HO-1 enzyme activity [90]. Genipin was effective at inhibiting LPS-induced nitric oxide (NO) release from cultured rat brain microglial cells and reduced the LPS-stimulated production of TNF-α, IL-1β, PGE_2_, intracellular ROS, and NF-κB activation [91]. In a study conducted by Koo *et al. *[92], genipin (**16**) exhibited a significant topical anti-inflammatory effect shown through inhibition of croton oil-induced ear oedema in mice. 

### 3.9 *Gentiana lutea *(Great yellow gentian)

*Gentiana lutea *L. (Gentianaceae) is a yellow flowering, perennial plant commonly found in the mountainous regions of central and southern Europe. It contains bitter-tasting secoiridoid glycosides; sweroside (**9**), swertiamarin (**12**) and gentiopicroside (**18**), which revealed cholagogue, hepatoprotective and wound-healing effects in pharmacological studies [93]. The genus *Gentiana* contains about 400 species and is the largest genus in the Gentianaceae family. The ethyl acetate extract of another species, *Gentiana striata* Maxim., showed anti-inflammatory activity in rats inflicted with rheumatoid arthritis. The paw volume in the treatment group (100 and 200 mg/kg) was significantly reduced compared to the prednisone control group and an increase in bodyweight was noted. There was a marked decrease in the PGE_2_ and NO levels. Six compounds including two iridoids, loganin (**8**) and sweroside (**9**), was isolated from this extract [94].

On investigating gentiopicroside analgesic activities and central synaptic modulation to the peripheral painful inflammation, Chen *et al.* [95] reported that gentiopicroside (**18**) produced significant analgesic effects against persistent inflammatory pain stimuli in mice. Systemic administration of gentiopicroside significantly reversed NR2B (NMDA Receptor 2B) over-expression during the chronic phases of persistent inflammation in Freund’s adjuvant-induced arthritis. The results suggested that the analgesic effect of gentiopicroside may be due to the modulation of glutamatergic synaptic transmission in the anterior cingulate cortex [95]. Park *et al.* [45] investigated the anti-inflammatory effect of hydrolysed iridoid products. No activity was recorded for swertiamarin (**12**) before β-glucosidase treatment. However, after treatment, H-swertiamarin (10 μM) significantly inhibited PGE_2_ formation in LPS-stimulated RAW 264.7 cells. For all the iridoids tested, activity was noted only after hydrolysis [45]. 

### 3.10 *Harpaphygotum procumbens* (Devil’s claw)

*Harpagophytum procumbens* (Burch.) DC. ex Meisn. ssp. *procumbens* (Pedaliaceae) is an important traditional medicine growing in the Kalahari region of southern Africa where it is consumed to treat rheumatism and arthritis as an analgesic. In addition, it is used as a cure for digestive disorders and as a general tonic or applied topically as an ointment to treat sores, ulcers and boils [96]. Many compounds have been isolated from *H. procumbens* including harpagoside, harpagide, procumbide and acteoside.

The anti-inflammatory mechanism of action of *H. procumbens* has not yet been satisfactorily elucidated. Jang *et al*. [97] found that the aqueous extract of *H. procumbens* suppressed PGE_2_ synthesis and nitric oxide production by inhibiting LPS-stimulated enhancement of the COX-2 and iNOS protein mRNA expression in L929 cells. These results suggested that *H. procumbens* exerted anti-inflammatory and analgesic effects probably by suppressing COX-2 and iNOS expressions. Some studies revealed that the efficacy of *H. procumbens* in reducing pain and inflammation associated with rheumatoid and osteoarthritis can be explained by its ability to block the production of inflammatory mediators such as PGE_2_ [93]. Kaszkin *et al*. [99] determined that the efficacy of *H.* *procumbens* was dependent on the ratios of harpagoside (**19**), harpagide (**20**), 8-coumaroylharpagide (**21**) and acteoside, also reported by Abdelouahab and Heard [100]. These compounds are believed to act synergistically or antagonistically in modulating the enzymes responsible for inflammation. However, most official monograph specifications are based on harpagoside content alone [100]. Miraldi *et al.* [101] proposed that this species seems to stimulate migration of interleukins and leucocytes to painful and inflamed joints. An extract of *H. procumbens* showed anti-inflammatory and antinociceptive effects in rats with Freund’s adjuvant-induced arthritis in both the acute and chronic phases [102]. 

Iridoid glycosides have been considered as the active constituents in *H.* *procumbens* and several studies have been completed to assess their anti-inflammatory activity. An *in vitro* study with Ca^2+^ ionophore A23187-stimulated human whole blood has revealed an inhibition of the biosynthesis of cysteinyl-leukotrienes and TBX_2_ by *H.* *procumbens* extracts as a function of their harpagoside (**19**) concentration [103]. Another whole-blood assay revealed that a high harpagoside-containing fraction inhibited COX-1 (37.2%), COX-2 (29.5%) and NO production (66%) [104]. Boje *et al*. [105] determined the ability of aqueous *H. procumbens* and *H. zeyheri* extracts and several iridoid and phenylethanoid glycosides to inhibit human leukocyte elastase *in vitro*. The release of elastase and other proteinases from macrophages and neutrophils is part of the inflammatory process and leucocyte elastase is found in inflamed tissue. Low IC_50_ values of 542 and 1012 μg/ml for *H. procumbens* and *H. zeyheri* water extracts, respectively, were noted. The acteosides were more active than the iridoid compounds. Pagoside (**22**) had an IC_50 _value of 260 μM and harpagoside (**19**) was less active with values in excess of 500 μg/ml-800 μg/ml [105]. Interestingly, Zhang *et al.* [44] found that the hydrolysed products of harpagide and harpagoside had a significant COX-2 inhibitory activity (2.5-100μM) where unhydrolysed harpagide and harpagoside did not. Therefore, the hydrolysis of the glycosidic bonds of harpagide and harpagoside by β-glucosidase is a prerequisite step for activity. A recent study by Gyourkovska *et al*. [106] reported anti-inflammatory action from extracts and preparations of *in vitro* cultured *H. procumbens*. Several fractions as well as pure compounds showed inhibitory action at a concentration of 1 mg/ml. The strongest activity was noted for verbascoside followed by the iridoid glycosides harpagide and harpagoside. Cytotoxicity assays revealed harpagide (1 mg/ml) as the most toxic compound which raises concern as it is present in commercial formulations [106]. 

The use of *H. procumbens* is approved in German Commission E and European Scientific Cooperative on Phytotherapy (ESCOP) monographs. In contrast to the other species in this review, more than 20 clinical studies using *H. procumbens* to treat lower back pain, osteoarthritis and arthritis have been published. In most cases, 4 weeks of treatment improved the pain index considerably. One such example of a study assessed the treatment of patients (n=75) with arthrosis of the hip or knee. For 12 weeks two tablets (400 mg aqueous extract/tablet) three times daily corresponding to a total of 2400 mg containing 50 mg iridoid glycosides calculated as harpagoside was administered. There was a strong reduction in the pain and symptoms of osteoarthritis. Pain scores (actual, worst, average, total) reduced by 22.6-25.8% and there was an improvement on clinical findings such as pain on palpation (45.5%), limitation of mobility (35%) and joint crepitus (25.4%). Adverse events such as dyspeptic complaints (2), sensation of fullness (1) and panic attack (1) were reported in only four patients [107]. A systematic review on the use of *H. procumbens* for osteoarthritis and lower back pain revealed effectivity on administration of powder, as well as ethanolic and aqueous extracts with doses containing 50-100 mg of harpagoside [108]. 

### 3.11 *Himatanthus sucuuba* (Sucuba; Janaguba)

*Himatanthus sucuuba* (Spruce ex Müll.Arg.) Woodson (Apocynaceae), a tree found in the Amazon rain forest, is a well-known remedy to treat various ailments. In Perú, an infusion from the stem bark has been used as a wound-healing agent, vermifuge laxative and hallucinogen, as well as for the treatment of tumours, boils, oedema and arthritis. In Brazil, infusions, decoctions, poultices and the latex of the bark are used in folk medicine for the treatment of gastritis, inflammation, anaemia, arthritis, verminosis and tumours [109]. The Caboclos in the Amazon use the dried stem bark for its analgesic and anti-tussive activities. Phytochemical studies led to the isolation of triterpene esters with anti-inflammatory activity and iridoids with cytotoxic activity [110]. Silva *et al.* [111] demonstrated that plumericin and isoplumericin may be associated with DNA damage. 

The latex, bark, leaves and roots have been shown to contain several iridoids namely plumericin, isoplumericin, plumieride, isoplumieride, 15-demethylisoplumieride, 15-dimethylplumieride, fulvoplumierin, b-dihydroplumericin and allamandin [110]. However, only plumericin (**23**) and isoplumericin (**24**) have been shown to possess anti-inflammatory activity [111]. 

### 3.12 *Kigelia africana* (Sausage tree)

*Kigelia africana *(Lam.) Benth. (Bignoniaceae) is a tropical tree, widely distributed in south, central and west Africa, used in folk medicine [112]. Traditionally, dried fruits are used to prepare emollients for topical application to treat psoriasis and eczema. Extracts from the root bark are used to treat venereal disease and naphtoquinones extracted from *K. africana *exhibited anti-trypanosomal, antimicrobial and anti-tumour activities against melanoma and renal carcinoma cells [112-113]. 

Analysis of a polar extract of the fruit confirmed the presence of verminoside (**25**), an iridoid glycoside, as a major constituent, as well as a series of polyphenols such as verbascoside. *In vitro* assays confirmed that verminoside had significant anti-inflammatory effects, inhibiting both iNOS expression and NO release in the LPS-induced J774.A1 macrophage cell line [114]. Using the carrageenan-induced paw oedema model in rats, as well as the acetic acid-induced writhing, hot plate and formalin-induced paw licking models in mice, Carey *et al*. [115] reports that the flower extract exhibited significant anti-inflammatory and analgesic activities at doses of 100, 200 and 400 mg/kg body weight in rats and mice. It is reported that verminoside (**25**) isolated from *K. africana*, exhibits cytotoxic activity in the concentration range of 70-355 μM. Additionally, apoptotic cell death due to verminoside was observed during histological analysis of the tested cell lines [116]. Hassan *et al.* [112] reports that* K. africana* extracts have significant wound-healing properties observing a rapid reduction in exudation and scab formation, classical signs of inflammation. The study also reported that the lethal dose of the leaf extract is greater than 3 g/kg; this suggests that *K. africana *is safe to be consumed for the treatment of various ailments. It is proposed that the mechanism of action of the leaf extracts may be due to its angiogenic and mitogenic potential leading to increased cellular proliferation and collagen synthesis [112]. This may assist in scar tissue formation and reperfusion of the injured site, thus resolving the sequela of inflammation. 

### 3.13 *Lamiophlomis rotata* (Duyiwei) 

*Lamiophlomis rotata* (Benth. ex Hook. *f*.) Kudô is a perennial Lamiaceous herb growing in the Qinghai-Tibet Plateau in northwestern China. For centuries *L. rotata* has been used as one of the traditional drugs in Tibetan, Mongolian and Naxi nations for detumescence, haemostasis, pain alleviation, blood circulation promotion and bone marrow regeneration [117]. 

Iridoids and flavonoids have been isolated from the aerial parts and roots of *L. rotata.* 8-*O*-Acetylshanzhiside methylester, 6-*O*-acetylshanzhiside methylester, shanzhiside methylester, 8-deoxyshanzhiside, sesamoside, loganin, penstemoside, phlomiol, 7,8-dehydropenstemoside, phloyoside I, phloyoside II, lamiophlomiside, phlorigidoside C, have all been isolated from the species in the past twenty years [117]. Only loganin (**8**), an iridoid glycoside, has been demonstrated to possess anti-inflammatory activity. In a recent study, the anti-inflammatory activity of *L. rotata* was determined by cotton pellet-induced granuloma formation in rats and xylene-induced ear oedema in mice. *Lamiophlomis rotata *injection at 0.45, 0.9 and 1.8 g/kg caused a dose-dependent inhibition of ear oedema induced by xylene equivalent to 43.87-68.16% protection and 13.26-43.33% protection in cotton pellet-induced granuloma at the doses of 0.225 and 0.45 g/kg, respectively. *Lamiophlomis rotata *injection increased phagocytosis by mouse peritoneal macrophages, and decreased the LPS-induced production of IL-1 [118]. 

### 3.14 *Mentzelia chilensis* (Blazing star; Angurate)

*Mentzelia scabra *subsp*. chilensis *Gay (Loasaceae) is a shrub widespread in South America (Peru, Chile and Bolivia). The aerial parts have cicatrizant, choleretic and antihelminthic properties [119]. A crude aqueous extract of *M. chilensis* showed anti-inflammatory activity in the carrageenan-induced rat-paw oedema model. Bioassay-guided isolation yielded the iridoid glycoside, mentzeloside (syn. deutzioside), as an active principle. Mentzeloside (**26**) showed a marked and significant dose-dependent inhibitory activity on carrageenan-induced rat-paw oedema with an ED_50 _of 40.4 µg/kg [120]. 

### 3.15 *Phillyrea latifolia* (Mock privet)

*Phillyrea latifolia* L. (Oleaceae) is mainly found in the Mediterranean coastal region. Dioscorides (1^st^ century BC) described the medicinal use of the leaves of privet, which were chewed to offer relief of oropharyngeal inflammation, while a decoction of the aerial parts was claimed to be active against burns and headaches [121]. Despite the known anti-inflammatory activity of the species, *P. latifolia *is cited rarely in ethnobotanical literature, the use of the leaves seems to survive still in some areas of South West Sardinia Latium and in Morocco for diuretic, spasmolytic and diaphoretic purposes. Moreover, the fruits of *P. latifolia* were once eaten in South Europe [121-122]. Diaz *et al.* [123] tested two iridoid glycosides, oleuropeoside (**27**) and ligustroside (**28**) isolated from *P. latifolia* for activity against the COX and 5-lipoxygenase (5-LOX) mediated arachidonate metabolism in calcium ionophore-stimulated mouse peritoneal macrophages and human platelets, and for their effect on cell viability. Oleuropeoside (IC_50_=47 μM) and ligustroside (IC_50_=48.53 μM) showed a significant effect on PGE_2_ release, with inhibition percentages similar to the reference drug indomethacin (IC_50_=0.95 μM). The effect on TXB_2_ release induced by calcium ionophore in human platelets was also investigated. Ligustroside (IC_50_=122.63 μM) showed a significant effect, although with less potency when compared to the reference drug ibuprofen (IC_50_=1.27 μM) [123]. 

### 3.16 *Picrorhiza kurroa* (Katurohini; Kutki)

*Picrorhiza kurroa *Royle ex Benth. (Scrophulariaceae) grows in the northwestern Himalayan region and is utilised in India as part of Ayurvedic medicine for the treatment of jaundice, indigestion, common fever, acute viral hepatitis and bronchial asthma. The root is considered to be a valuable bitter tonic, cholagogue and laxative in small doses. In addition, it is useful to treat gastrointestinal and urinary disorders, leukoderma, snake bite, scorpion sting and inflammatory disorders. Pharmacological studies have revealed hepatoprotective, anti-inflammatory, anti-asthma, immunostimulatory and free radical scavenging activities [124]. *Picrorhiza kurroa *contains iridoid glycosides such as picroside-I; picroside-II; picroside-IV, picroside-V, kutkoside, minecoside, veronicoside, 6, *O*-*trans*-ferulloyl catalpol, 6-*O*-*cis*-ferulloyl catalpol, pikuroside, as well as cucurbitacin glycosides and phenolic compounds [125].

A 50% ethanolic extract of *P. kurroa *was found to stimulate the cell-mediated and humoral components of the immune system as well as phagocytosis in experimental animals [124]. Anti-inflammatory activity was observed for kutkin (a mixture of kutkosides and picrosides) in experimentally induced arthritis, oedema, vascular permeability, and leukocyte migration in rodents [126]. Aucubin (**14**) was also shown to inhibit phorbolester-induced oedema in mice ears, while catalpol (**15**) and picroside II (**29**) were not active (100 mg/kg p.o.). The latter iridoids showed only minor anti-inflammatory effects upon topical administration [43]. Moderate anti-inflammatory activity of picroside II (**29**), when administered topically, was confirmed later, while pikuroside was ineffective [127]. Picrosides II (**29**), III (**30**), V (**31**), 6-feruloylcatalpol (**32**) and minecoside (**33**) moderately inhibited chemiluminescence generated by activated polymorphonuclear neutrophils (PMNs); scavenging effects of these compounds were excluded. Picroliv, an iridoid glycoside mixture containing picroside I (**34**) and kutkoside (**35**), was shown to be moderate superoxide scavengers, while kutkoside alone showed only weak activity [128]. Furthermore, Picroliv (**34** & **35**) protected cells against hypoxia, enhanced the expression of vascular endothelial growth factor and hypoxia inducible factor-1, selectively inhibited protein tyrosine kinase activity, and reduced protein kinase C [129]. 

### 3.17 *Plantago asiatica* (Chinese Plantain; Arnoglossa)

*Plantago asiatica *L. belongs to the Plantaginaceae and has a history of use for the treatment of many conditions such as bronchitis, diarrhoea, constipation and wounds. This herbal medicine has also been shown to exhibit antileukaemia, anticarcinoma and antiviral activities, as well as activities which modulate cell-mediated immunity. The seeds of *P. asiatica *were regarded as a traditional medicine in ancient Chinese medical literature. Its chemical components, including phenylethanoid glycosides, phenolics and various polysaccharides have been widely studied. Aucubin (**14**) is an iridoid glycoside that has been isolated from *P. asiatica* [130]. Shim *et al.* [83] investigated the effects of 0.1% aucubin on oral wound-healing. Re-epithelisation and matrix formation of the aucubin-treated group occurred earlier and the number of inflammatory cells was fewer than that of the control group. Thus aucubin may be applied as a topical agent to accelerate the healing of oral wounds. 

RAW 264.7 cells were used by Kyoung and Chang [131] to elucidate the mechanism of action of aucubin and its hydrolysed product produced by β-glucosidase treatment. The hydrolysed product suppressed m-RNA expression for both the TNF-α and subsequent TNF-α protein, whereas aucubin did not. This occurred in a dose-dependent manner with an IC_50 _value of 9.2 μM. Additionally, the hydrolysed product blocked both I-κBα degradation and the translocation of NF-κB from the cytosol fraction to the nuclear fraction (55% inhibition). Treatment with hydrolysed aucubin did not affect the intracellular level of cAMP formed by forskolin treatment in human monocytes U937 culture, implying that there is no influence on the cAMP level in other cell systems [131]. 

### 3.18 *Rehmannia glutinosa* (Chinese foxglove) 

*Rehmannia glutinosa *Steud. which belongs to the Scrophulariaceae is a medicinal herb that is believed to maintain haemostasis, promote blood circulation and improve kidney function. Traditionally, the steamed root of the plant is used to treat allergic inflammatory diseases such as contact dermatitis and rhinitis. Mite allergen-treated NC/NGa mice were treated with an ethanol extract to observe its effect on skin inflammation. *In vitro* tests revealed a decrease in the mRNA expression of Il-4, TNF-α, VCAM-1 and ICAM-1 as well as a decrease in dermal thickening and infiltration by inflammatory cells. In addition, ear thickness and serum histamine levels were significantly reduced *in vivo* [132]. Catalpol (**15**), an iridoid glycoside, isolated from the species, has been verified to be neuroprotective and may be a potential agent for the treatment of neurodegenerative disease [133]. Glia-mediated inflammation is significant in the pathogenesis of Alzheimer's disease. Catalpol (**15**) reduces the release of inflammatory factors including TNF-α, ROS, NO and iNOS, which accelerates the progression of Alzheimer's disease. 

### 3.19 *Russelia equisetiformis* (Firecracker, coral plant)

*Russelia equisetiformis* (Schltdl. & Cham.) belongs to the Scrophulariaceae family. This small tree, native to tropical America, is commonly used for ornamental and slope protection purposes. It is reported that the plant is traditionally used in Nigeria for the treatment of diabetes, leukemia and pain and inflammation. Scientific studies have revealed analgesic, anti-inflammatory, antimicrobial, free radical scavenging and membrane-stabilising activity [134-136]. Whole organic plant extracts and isolated phenolic compounds exhibited antinociceptive activity in male Swiss mice [134] in both the writhing and tail-flick tests. Oral administration of a methanol extract (100 mg/kg) showed significant inhibition or reduction in both egg albumin and agar-induced paw oedema in female albino Wistar rats, comparable to indomethacin (5 mg/kg). The authors suggested a possible mechanism of action through inhibition of pro-inflammatory mediators. In addition, free radical scavenging and anti-oxidant properties were found [135]. A new iridoid glycoside, 10-*O*-cinnamoyl sinuatol (**36**), was isolated from a leaf extract of *R. equisetiformis* together with 24 known compounds including iridoid, phenyl propane, lignin and flavonoid glycosides as well as phenyl ethanoids. Mild alkaline hydrolysis of 10-*O*-cinnamoyl sinuatol yields sinuatol which has previously been isolated from *Verbascum sinuatum*. The isolated compounds, including rehmaglutin B (**37**), catalpol (**15**), 6-*O*-*cis*-p-coumaroylcatalpol, and verminoside (**25**) amongst others were assayed for their inhibitory activity towards NO production *in vitro* using RAW 264.7 cells. The iridoid compounds verminoside (±8%) (**25**) and rehmaglutin B (70%) (**37**) showed NO inhibitory activity at 100 µM and rehmaglutin B (IC_50_ = 52.8±2.0 µM) inhibited NO production more strongly than the control (L-NMMA) [136]. 

### 3.20 *Scrophularia auriculata* (Water figwort)

*Scrophularia auriculata* L. ssp. *pseudoauriculata* (Scrophulariaceae) is a Mediterranean plant used in folk medicine to treat inflammatory skin diseases. Two iridoid glycosides, scropolioside A (**38**) and scrovalentinoside (**39**), isolated from the species have been described as anti-inflammatory and immunomodulatory compounds. Scropolioside A (**38**) showed anti-inflammatory properties against different experimental models of delayed-type hypersensitivity. Scropolioside A (**38**) reduces oedema both *in vivo* and* in vitro. In vivo, *it reduced oxazolone-induced oedema by 79% (0.5 mg/ear) and *in vitro*, by 43% (10 mg/kg) in ovine red blood cells. *In vivo* it reduced both oedema formation and cell infiltration whereas *in vitro* it reduced the proliferation of activated T-lymphocytes with an IC_50_ value of 67.74 μM [39]. Scropolioside A (**38**) inhibited the production of PGE_2_, leukotriene B_4_, NO, IL-1β, IL-2, IL-4, TNF-α and interferon-γ, but has no effect on the production of IL-10. Moreover, Scropolioside A (**38**) modified the expression of both nitric oxide synthase-2 and COX-2, as well as the activated NF-κB in RAW 264.7 macrophages [40]. 

### 3.21 *Scrophularia deserti* (Afinah, Zetah, Maseelah)

*Scrophularia deserti *Delile (Scrophulariaceae) is the most common figwort found in Kuwait and is fairly abundant in limestone-rich areas [137]. It is also found in Saudi Arabia, where it is used as an antipyretic, a remedy for kidney diseases, cardiotonic, hypoglycaemic, as a diuretic in typhoid fever, to treat galactorrhoea and inflammation of the mouth, lungs, large intestine, bladder, and heart, as well as a remedy for tumours, abscesses, cancer of the lung, goiter, and aching bones [138]. Ahmed *et al. *[138] isolated five iridoid glycosides, including two “new” compounds scropolioside-D_2_ (**40**) and harpagoside-B, from the aerial parts of *S. deserti *which were found to possess significant antidiabetic and anti-inflammatory activity, respectively. However, in 2007 Jensen and co-workers [139] demonstrated that the structure proposed as harpagoside-B by Ahmed *et al*. [138] is structurally identical to harpagoside (**19**). When tested on carrageenan-induced rat-paw oedema at a dose of 10 mg/kg, harpagoside (=harpagoside-B) and the iridoid diglycoside koelzioside (**41**) were the most active compounds and showed a decrease in oedema by 30% and 26% respectively after 3 h [138]. These results allowed the researchers to speculate on possible structure activity relationships (SAR) as the two most active compounds both has cinnamoyl moieties in place of acetyl groups. 

### 3.22 Scrophularia frutescens

The Scrophulariaceae is represented by several iridoid-containing genera such as *Scrophularia *with many species accumulating high levels of harpagoside (**19**). This iridoid glycoside has found wide use in clinical practice for the treatment of pain in the joints and lower back for its neuroprotective and anti-inflammation activities [140]. An aqueous and methanolic extract together with harpagoside (**19**) obtained from *Scrophularia frutescens* were tested for anti-inflammatory activity using the rat-paw oedema assay. The aqueous extract exhibited a low yet significant anti-inflammatory effect on carrageenan-induced oedema, while the methanolic extract produced lower anti-inflammatory activity. The activity of the isolated harpagoside (**19**) was negligible in the same assay leading the authors to conclude that the observed anti-inflammatory activity may not be ascribed to harpagoside alone [141]. This finding concurs with Zhang *et al.* [44] who reported that harpagoside had no effect on COX-1/2 enzyme activity, TNF-α release and NO production *in vitro*. However, after treating harpagoside with β-glucosidase, the hydrolysed product, H-harpagide showed a significant inhibitory effect on COX-2 at 2.5-100 µM in a concentration-dependent manner. It is noteworthy that this hydrolysed product has a backbone similar to commercially available COX-2 inhibitors such as celecoxib. Although this review is acutely focused on iridoids one needs to be cognisant of other phytochemicals which may elicit an anti-inflammatory response, either alone or in combination with iridoids. 

### 3.23 *Scrophularia scorodonia* (Balm-leaved figwort)

*Scrophularia scorodonia* L. is widespread in the southwestern parts of Spain and in the northwest of Africa [142]*. *Seven iridoid glycosides isolated from different extracts of *S. scorodonia*, namely, aucubin (**14**), harpagoside (**19**), harpagide (**20**), 8-acetylharpagide (**42**), scorodioside (**43**), scropolioside B (**44**) and bartsioside (**45**), were evaluated for their *in vitro* anti-inflammatory activity in cellular systems generating COX and LOX metabolites. Most compounds assayed did not exhibit a significant effect on PGE_2_ and LTC-4 release from calcium ionophore-stimulated mouse peritoneal macrophages. In the LTC-4 assay, only aucubin (**14**) showed a significant effect, with an IC_50 _value of 72 µM. Harpagoside (**19**) and harpagide (**20**) also inhibited release of LTC-4. The release of PGE_2_ by mouse peritoneal macrophages stimulated with calcium ionophore was inhibited by harpagoside (**19**) and 8-acetylharpagide (**42**). Most iridoids assayed showed a significant effect on TXB_2_ release from calcium ionophore-stimulated human platelets, with inhibition percentages slightly lower than the reference drug ibuprofen. Harpagide (**20**), scorodioside (**43**) and scropolioside B (**44**) had no significant effect on TXB_2_ release. These findings indicate that selective inhibition of the TX-synthase enzyme may be the primary target of action of most of these iridoids, and one of the mechanisms through which they may exert their anti-inflammatory effects [143]. 

### 3.24 *Sideritis perfoliata* (Mountain tea)

*Sideritis perfoliata *L. subsp. *perfoliata *is a plant widely used in folk medicine in Greece mainly for its anti-inflammatory, anti-rheumatic, antiulcer, digestive and vasoprotective properties. The genus *Sideritis* comprises more than 150 perennial and annual species distributed in the Mediterranean area. The infrageneric taxonomy is complex due to hybridisation events. Traditionally, it is consumed as a herbal tea, used as a flavouring agent and extensively used in folk medicine as an anti-inflammatory, anti-ulcerative, antimicrobial, vulnerary, anti-oxidant, antispasmodic, anticonvulsant, analgesic and carminative. Phytochemical studies of the polar extracts afforded four flavonoid glycosides, four phenylpropanoic glycosides, caffeic acid and one iridoid, ajugoside (**46**) [144]. These compounds were evaluated for their anti-oxidant activity (DPPH assay) as well as for their anti-inflammatory activity using the soybean lipoxygenase bioassay. All extracts and isolated compounds showed significant anti-oxidant and inhibitory activity against soybean lipoxygenase. Ajugoside (**46**) however, was one of the least active molecules tested [144].

### 3.25 *Stachytarpheta cayennensis* (Brazillian tea)

*Stachytarpheta cayennensis* (Rich.) Vahl (Verbenaceae) is widely spread throughout tropical and suptropical America where it is extensively used in Brazilian folk medicine as an anti-inflammatory, analgesic, antipyretic, hepatoprotective, laxative and to treat gastric disturbances. This species has an extensive ethnobotanical history of use by the Caboclo inhabiting the Marajo island in the Amazon delta. Several of the traditional uses allude to anti-inflammatory properties and in the early nineties *Stachytarpheta cayennensis *was one of four species recommended by the United Nations Development Programme for possible pharmaceutical development [145]. A tea prepared from the dried leaves or a tincture is used to treat gastrointestinal diseases and pain. Topical application of the macerated leaves and roots is used to treat skin wounds [146]. Penido *et al. *[147] assessed the anti-inflammatory properties of ethanolic extracts of *S. cayennesis*. Chromatographic analysis of the crude ethanolic extract revealed high concentrations of the iridoid ipolamiide (**47**). The oral administration of the extract (100 mg/kg) to Swiss albino mice failed to inhibit paw oedema and pleural exudation induced by carrageenan and zymosan. Ipolamiide (**47**) inhibited total leukocyte accumulation into the pleural cavity 4 and 24 h after the intrathoracic injection of carrageenan, due to the inhibition of neutrophil and mononuclear cell influx. Ipolamiide also selectively inhibited neutrophil influx. A structurally related compound, lamiide (**5**) which is devoid of the C_7_-OH group, was also reported to possess dose-dependent anti-inflammatory activity (79%) against carrageenan-induced oedema [56] in similar experimental protocols and at the same dose levels. 

Schapoval *et al. *[146] investigated the alcoholic and *n*-butanolic extracts of dried leaves of *S. cayennensis *in anti-inflammatory and antinociceptive models. Intraperitoneal pretreatment with the dried extracts at doses ranging from 100 to 200 mg/kg, significantly inhibited carrageenan-induced inflammation. The active extracts were fractionated and monitored using the same bioassay. The iridoid ipolamiide (**47**) and the phenylethanoid glycoside acteoside were isolated from the active fraction and showed an inhibitory effect on histamine- and bradykinin-induced contractions of guinea-pig ileum. Ipolamiide (**47**) and acteoside also showed *in vivo* anti-inflammatory activity when administered orally to rats mainly in the fourth hour after the administration of the phlogistic agent, 70.22% and 93.99%, respectively. An adverse effect of many NSAIDs is gastric disturbances. As a secondary objective the authors explored the rationale for the traditional use of *S. cayennensis *to treat gastric disorders and ulcers. The *in vivo* model proved that an extract protected the gastric mucosa against damage caused by diclofenac, a classic NSAID [148].

### 3.26 Syringa species

The term folium syringae is used to collectively refer to the leaves of several species of *Syringa* (Oleaceae) such as *S. oblata*, *S. ditaata* *S. vulgaris* and *S. chinensis*. These species are popular ornamental and holticultural bushes cultivated in Eurasia and North America. Folium syringae have been used as a folk medicine to treat inflammatory diseases, especially intestinal inflammations such as acute enteritis, icteric hepatitis, acute mastitis, bacillary dysentery and upper respiratory tract infection in China. Two folium syringae preparations have been listed in the Drug Standard of the Ministry of Public Health of China. Phytochemical studies demonstrated that iridoid glycosides are the main active fraction of the leaves. The iridoid glycoside, syringopicroside (**48**), is the main active constituent and has been shown to possess anti-inflammatory, broad-spectrum antimicrobial, antiviral, and immune enhancement effects [149-150]. 

Liu and Wang [150] investigated the anti-inflammatory effects of the iridoid glycoside fraction of folium syringae leaves on TNBS-induced colitis in rats. Ulcerative colitis was induced through rectal administration of TNBS. The iridoid glycoside fraction (80, 160, 240 mg/kg) was orally administered (10 ml/kg) twice daily 24 h after colitis was established. Treatment with the iridoid glycoside fraction (containing 55.74% syringopicroside) suppressed pathological symptoms caused by TNBS and dose-dependently inhibited the elevated level of NO, in fact, the 240 mg/kg dose exhibited better therapeutic effects compared to the positive control salicylazosulfapyridine (SASP). Myeloperoxidase (MPO) activity was reduced and malondialdehyde (MDA) levels were suppressed indicating that this fraction successfully scavenges oxidative free radicals. In addition, the fraction effectively inhibited the protein and mRNA expressions of the pro-inflammatory cytokines NF-κBp65, TNF-α and IL-6 in a dose-dependent manner [150]. 

In a follow up study, Liu and Wang [157] determined that this fraction dose-dependently depressed the levels of the pro-inflammatory cytokines TNF-α and IL-8, the inflammatory protein COX-2, and TGF-1β levels in the colon tissues. It significantly blocked NF-κB signaling by inhibiting IκBα phosphorylation/ degradation and IKK-β (inhibitor of NF-κB) activity. In addition, it down-regulated the protein and mRNA expressions of Fas/FasL, Bax and caspase-3, and activated Bcl-2 in intestinal epithelial cells. Administration of this syringopicroside-rich fraction resulted in marked protective effects on dextran sulfate sodium (DSS)-induced colitis through inhibition of intestinal epithelial cell (IEC) apoptosis and blockade of the NF-κB signal pathway [151].

### 3.27 *Verbascum species* (Mullein)

Extracts, decoctions and infusions prepared from *Verbascum* species (commonly known as “mullein”) have an illustrious history of traditional use, especially for the treatment of respiratory disorders. It is believed that the extracts exert their activity through expectorant, mucolytic and demulcent properties. *Verbascum *flowers are boiled in milk and are applied externally for pruritic conditions affecting the urogenital organs. Several species are specifically used in Turkey and said to be mildly diuretic and to have a soothing and anti-inflammatory effect on the urinary tract, in addition to acting as a mild sedative [152-153]. Oil prepared from the flowers is used to soothe earache, and can be applied externally to treat eczema and other types of inflammatory skin conditions [154]. 

A methanolic extract of the flowers of *Verbascum lasianthum* Boiss. (Scrophulariaceae) was shown to possess significant inhibitory activity in the carrageenan-induced hind paw oedema model and in *p*-benzoquinone-induced writhings in mice. Through bioassay-guided fractionation several compounds, including aucubin (**14**), catalpol (**15**), geniposidic acid (**17**) and ajugol (**49**) were isolated which have been shown in previous studies to have anti-inflammatory activity. Orally administered ucubin (**14**) was found to possess significant antinociceptive and anti-inflammatory activities without inducing any apparent acute toxicity or gastric damage [155].

*Verbascum mucronatum* Lam. is used as haemostatic in Turkish folk medicine and exhibits anti- inflammatory, antinociceptive and wound healing properties [152]. The aqueous extract (200 mg/kg) of *V. mucronatum *produced an anti-inflammatory effect comparable to indomethacin (10 mg/kg) in the carrageenan-induced hind paw oedema model [156]. Through assay-guided fractionation, catalpol (**15**), ajugol (**49**), lasianthoside (**50**), picroside IV (**51**) and two saponins, ilwensisaponin A and C verbascoside were isolated. In the same study the *in vivo* wound-healing activity of *V. mucronatum* was evaluated by linear incision and circular excision experimental models and subsequent histopathological analysis. The healing potential was comparatively assessed with a reference ointment Madecassol^® ^which contains a 1% extract of *Centella asiatica*. *Verbascum mucronatum* was found to accelerate the wound-healing processes (inflammation, proliferation and re-modeling), confirming the widespread use of this species in traditional medicine [156]. 

Tatli and Akdemir [157] isolated ajugol (**49**) and picroside IV (**51**) and several other phenolic compounds from *Verbascum pterocalycinum* var. *mutense *Hub.-Mor. These compounds together with saponin glycosides were investigated for anti-inflammatory and antinociceptive properties [158]. A dose-related anti-inflammatory and antinociceptive response was obtained at 100 and 200 mg/kg. Although the activities of ajugol and picroside IV were found to be insignificant, the saponins, ilwensisaponin A and C showed notable activity without inducing any apparent acute toxicity or gastric damage.

### 3.28 *Verbena officinalis* (Vervain)

Although native to Europe, *Verbena officinalis* L. (Verbenaceae) has become naturalised in many countries and is especially renowned in China as a medicine to treat fever and to detoxify the body. The plant is said to promote blood circulation, remove blood stasis and induce diuresis. It has also been used in folk medicine as a diuretic, expectorant and anti-rheumatic. In Navarra, Spain, it is used extensively in traditional medicine, especially topically due to anti-inflammatory effects [159]. The main components of *V. officinalis* are iridoids, phenylpropanoids, flavonoids, and terpenoids [160]. 

The effects of a cream containing a 50% methanolic extract of *V. officinalis*, incorporated in various concentrations (1%, 1.5%, 2%, 2.5%, 3%), was tested on a carrageenan-induced oedema and formalin testing animal model. Piroxicam gel and methyl salicylate ointment were used as positive controls for anti-inflammatory and analgesic activity respectively. The cream formulation reduced inflammatory oedema dose-dependently, with the 3% formulation possessing activity similar to piroxicam gel. In the analgesia test the cream dose-dependently exerted topically induced analgesia, although not to the same extent as methyl salicylate. The active constituents believed to be responsible for the antinociceptive and anti-inflammatory activity are the iridoids, caffeoyl derivatives and flavonoids [161]. Using a mouse model, Calvo and co-workers [162] administered a 50% methnolic extract prepared from the leaves of *V. officinalis* and the isolated iridoid glucoside verbenalin (**52**) topically and orally to assess anti-inflammatory activity. In the TPA-induced ear inflammation assay the extract and verbenalin showed superior activity over oral administration in the carrageenan-induced rat-paw oedema model [162]. 

### 3.29 *Veronica anagallis-aquatica* (Water speedwell; Brook pimpernel)

*Veronica anagallis-aquatica* L. (Plantaginaceae) grows naturally in the Batiste Springs in the state of Idaho, USA. *Veronica anagallis-aquatica *is used for the treatment of influenza, haemoptysis, laryngopharyngitis and hernia. Traditionally, the aerial parts of the plant are boiled in milk to obtain a poultice which is applied to the abdomen to treat pain or in Anatolian regions the warm aqueous extract is used as a bath remedy to alleviate rheumatic pain [163]. Through bioassay-guided fractionation procedures, the major compounds isolated from the aerial parts of *V. anagallis-aquatica* were isolated. They include the calpol-derived catalposide (**16**) veronicoside (**53**) and verproside (**54**). These compounds were found to have a significant inhibitory effect on carrageenan-induced mouse-paw oedema. Doses were estimated according to the molar ratios of the iridoid glycosides in the biologically active fraction, as well as in standard doses of 250 and 500 mg/kg [163]. Catalposide (**16**) significantly inhibited the production of NO in LPS-stimulated RAW 264.7 macrophages in a dose-dependent manner. RT-PCR and Western blot analyses demonstrates that catalposide also suppressed the expression of the iNOS gene and protein and inhibited the activation of LPS-induced NF-κB [66]. Recio *et al.* [43] suggested a structure-activity relationship for topical anti-inflammatory activity of iridoid glycosides. OH-substitution at C_5_, unsaturation at C_7_ - C_8_, methyl substitution of carbonyl C_11_ and the integrity of the cyclopentane ring were essential for higher activity. In their study, Küpeli *et al.* [163] reported that esterification from C_6_ with benzoic acid (as in veronicoside) or phenolic acids (*p*-hydroxybenzoic acid in catalposide and protocatechic acid in verproside) provided a significant increase in anti-inflammatory activity in higher doses.

### 3.30 *Vitex peduncularis* (Boruna; goda)

*Vitex peduncularis *Wall*. *ex Schauer (Verbenaceae) is found in India and Khasia Terai. The bark is used for making an external application for chest pains. An infusion of the leaves, root bark or young stem bark is indicated for malarial type fever, especially in black water fever [164]. In 1921 the British Medical Journal reported an observational study where patients were given infusions of the leaves prepared from *V. peduncularis* to alleviate malaria associated fever. This lead was taken from ethnomedicinal uses of Aboriginal tribes. In all cases the infusion alleviated seized the fever. 

A new iridoid, pedunculariside (**55**), together with agnuside (**56**) were isolated from the butanol extract of *V. peduncularis *stem bark. In a murine cell-based assay both pedunculariside and agnuside showed selective inhibition of COX-2, with IC_50_ values of 0.15 mg/ml and 0.026 mg/ml, respectively. This selectivity to COX-2 receptors is essential in order to circumvent the notorious side-effects commonly associated with NSAIDs. Neither of the compounds exhibited cytotoxicity against vero cells [165]. 

## CONCLUSIONS

Inflammation is a complex process involving numerous mediators affecting the majority of organs in the body. An article referring to inflammation as “*The Secret Killer*” published in the popular *Time Magazine*, eloquently describes the link between inflammation and various disease conditions: “It destabilises cholesterol deposits in the coronary arteries, leading to heart attacks and potentially even strokes. It chews up nerve cells in the brains of Alzheimer's victims. It may even foster the proliferation of abnormal cells and facilitate their transformation into cancer. In other words, chronic inflammation may be the engine that drives many of the most feared illnesses of middle and old age.” [166]. Certainly, research has shown that this is indeed the case, which boldly emphasises the urgent and relevant need which exists to discover novel anti-inflammatory agents to treat the various disease conditions which manifest themselves through the inflammatory process. 

The data presented in this review has highlighted the following concepts;

1. Almost all of the species discussed in this review have a rich history of traditional use in treating inflammatory conditions, whether acute or chronic. Ethnobotanical leads have provided mankind with many new medicines and this is also true in the case of inflammation as highlighted in this review. The data presented here for several species lends credence and value to indigenous knowledge systems, a healing modality, which has been in existence far longer than any pharmaceutical company. The challenge however remains to generate sufficient scientific data which will allow this indigenous knowledge to be transformed into consumer products.
2. In order to provide both focus and structure to the review only iridoids have been discussed. Convincing pharmacological data is presented for several iridoids which provides evidence that this group of phytochemicals is active in treating inflammation-related disorders. However, several examples have been documented suggesting that the iridoids do not act in isolation but may exert their activity when acting in synergy with other molecules e.g. acteoside, which often co-occurs with iridoids. Despite the fact that many leads are taken from traditional healing practices scientists still give preference to a reductionist approach in the drug discovery process. Phytosynergy studies on iridoids (and other compounds) may present an exciting and rewarding research opportunity to better understand and unravel the complex interactions which may exist in treating inflammation.
3. The inflammation process is complex and numerous mechanisms of action are possible. Therefore it is important to note that if an extract or compound is not active in one assay, it does not imply that it possesses no anti-inflammatory activity. It may still exert anti-inflammatory activity at another level in the inflammation cascade such as through suppressing the expression of pro-inflammatory mediators like TNF-α. It may also be beneficial for a compound to selectively inhibit inflammation through action on COX-2 which would then negate the infamous gastric side-effects commonly associated with NSAIDs through COX-1 inhibition. Two iridoids isolated from *V. peduncularis* namely pedunculariside and agnuside showed selective inhibition of COX-2. In addition, *S. cayennensis* was shown to protect the gastric mucosa against damage caused by the classic NSAID diclofenac in addition to exhibiting anti-inflammatory activity. Exploring the possible interaction between natural products and conventional medicine could certainly be of scientific and therapeutic interest.
4. Though *in vitro* data contributes greatly towards elucidating the mechanism of action of any compound / extract, *in vivo* data remains crucial. For some species discussed only *in vitro* data is available. Even where *in vivo* studies have been done in animals, the process of developing anti-inflammatory drugs from botanical origin seems to terminate at this point, as literature on clinical data (*in vivo* human) remains scarce. *Harpagophytum procumbens* has been tested in humans, as highlighted in this review, with great success. Several *in vitro* studies have shown that the hydrolysed products of various iridoids showed better activity. In the case of harpagide and harpagoside, hydrolysis of the glycosidic bonds by β-glucosidase is a pre-requisite for activity. Therefore, it is important to investigate this clinically as well as oral administration would cause this necessary conversion. The prodrug effects will clearly not be detected *in vitro*. Although various acute toxicity and histopathology studies have shown that many of the extracts and/or iridoids mentioned in the review are seemingly well tolerated even in high doses, toxicity data is still lacking for many species discussed.
5. Elucidating structure-activity relationships presents a challenging, yet potentially rewarding opportunity for producing more active compounds derived from the iridoid scaffold. There are several examples where substitutions resulted in superior anti-inflammatory activity. Minor changes in chemical structure such as introducing a hydroxyl group resulted in an increase in topical anti-inflammatory activity in some cases. As already mentioned, the activity of iridoid glycosides increased after hydrolysis. Esterification from C_6_ with benzoic acid or *p*-hydroxybenzoic caused a significant increase in anti-inflammatory activity in higher doses in the case of veronicoside and catalposide, respectively. Not only with these modification optimise the activity of the molecule but may also influence the pharmacokinetics of the molecules. This group of natural products presents the medicinal chemists with a plethora of opportunities which hitherto remains largely neglected.

It is evident that this class of compounds presents a fascinating opportunity for further research. There are numerous species from which iridoids have been isolated that has as yet not been tested for anti-inflammatory activity. In addition, the general increase in lifestyle-related conditions in the population that is caused by inflammation provides endless research opportunities as yet unexplored.

## Figures and Tables

**Fig. (2):**
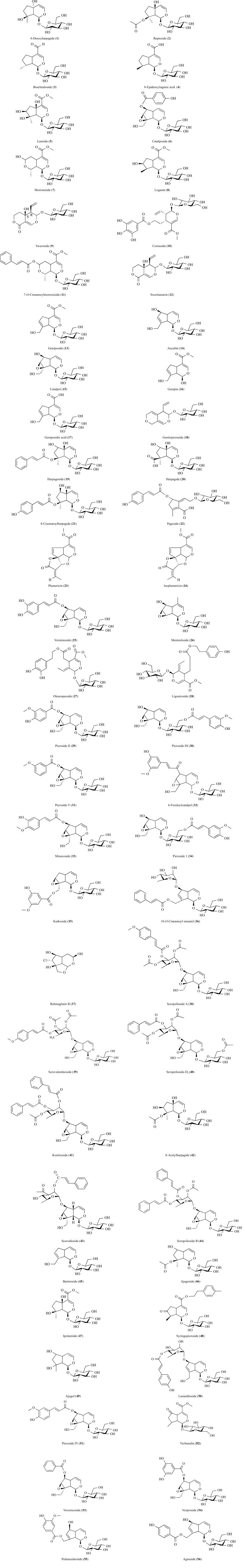
Chemical structures of selected isolated iridoid glycosides.

**Table 1.** Summary of Iridoid-Containing Plant Species Investigated for Anti-Inflammatory Properties

Species Name	Plant Part(s) Used	Isolated Iridoid Glycoside	Common Uses / Biological Activity	*In vitro* Studies	*In vivo* Studies	Proposed Mechanism of Action	References

*Ajuga bracteosa* Wall. ex Benth. (Lamiaceae)	Whole plant	6-deoxyharpagide (**1**); raptoside (**2**)	Hepatitis; pneumonia; bone disease	Yes	Yes	COX-2 inhibition.	[46-48]

*Boschniakia rossica* (Cham. & Schltdl.) Standl. (Orobanchaceae)	Whole plant	Boschnaloside (**3**); 8-epideoxyloganic acid (**4**)	Antisenile agent	Yes	Yes	TNF-α, iNOS, COX-2 inhibtion.	[49-53]

*Bouchea fluminensis* (Vell.) Moldence (Verbenaceae)	Aerial parts	Lamiide (**5**)	Bowel stimulator; anti-inflammatory agent	Yes	Yes	Inhibition of phospholipid peroxidation and free radical scavenging activity.	[56-58]

*Catalpa ovata *G. Don. (Bignoniaceae)	Stem	Catalposide (**6**)	Anti-inflammatory agent	Yes	Yes	Inhibition of the production of TNF-α and NO with significant decreases in mRNA levels of TNF-α and inducible NO synthase.	[64-67]
						Attenuates the induction of intestinal epithelial pro-inflammatory gene expression and reduces the severity of trinitrobenzene sulfonic acid-induced colitis in mice.	
						Inhibits the production of tumour necrosis factor-α (TNF-α), interleukin-1 (IL-1), and interleukin-6 (IL-6), and the activation of nuclear factor-B.	

*Cornus officinalis *Siebold et Zucc (Cornaceae)	Fruit	Cornel iridoid glycoside (CIG); morroniside (**7**); loganin (**8**); cornuside (**10**); 7-O-cinnamoyl-morroniside (**11**)	Anti-inflammatory and haemostasis-promoting agent	Yes	Yes	Ihibition of IL-1, IL-6, TNF-α, PGE_2_, iNOS and E-selectin expression, NO production, NFκB and COX-2.	[68-76]
						Suppression of ICAM-1, VCAM-1 and MCP-1.	

*Enicostema axillare* (Lam.) A.Raynal (Gentianaceae)	Whole plant	Swertiamarin (**12**)	Diabetes mellitus; rheumatism; ulcers; hernia; swelling; itching and anti-inflammatory agent	Yes	Yes	Anti-oxidant and hepatoprotective.	[45; 77-79]
						Inhibits TBX_2_.	

*Eucommia ulmoides* Oliv. (Eucommiaceae)	Bark and leaves	Aucubin (**14**); Genipin (**16**);	Diabetes mellitus; hypertension; anti-oxidant and anti-inflammatory agent	Yes	Yes	Concentration-dependent inhibition on lipid peroxidation induced by Fe^2+^/ascorbate.	[80-83]
						Concentration-dependent inhibition of NO production and iNOS expression upon stimulation by lipopolysaccharide / interferon-g.	
						Blockage of lipopolysaccharide indicating that it exhibits inhibitory effect on NO production through the inhibition of NFK-B activation.	
						Promotes wound healing.	

*Gardenia jasminoides* J.Ellis (Rubiaceae)	Fruit	Geniposide (**13**); Genipin (**16**)	Sedative; diuretic; cholagogue; antiphlogistic; anti-inflammatory; anti-oxidant and anti-thrombotic agent	Yes	Yes	Inhibition of lipopolysaccharide-stimulated iNOS expression or COX-2 promoter activity.	[84-92]

*Gentiana lutea *L. (Gentianaceae)	Roots	Gentiopicroside (**18**)	Gastric stimulation and anti-inflammatory agent	No	Yes	Reversal of NR2B over-expression during the chronic phases of persistent inflammation caused by hind paw administration of complete Freund’s adjuvant.	[45; 93-95]

*Harpagophytum procumbens* (Burch.) DC. ex Meisn. subsp. *procumbens* (Pedaliaceae)	Secondary tubers	Harpagoside (**19**); harpagide (**20**); 8-coumaroyl-harpagide (**21**); pagoside (**22**)	Rheumatism; arthritis; sores; ulcers and boils	Yes	No	Inhibition of the biosynthesis of cysteinyl-leukotrienes and TBX_2._	[44; 96-108]
						Suppression PGE_2_ synthesis and NO production by inhibiting LPS-stimulated enhancement of the COXe-2 and iNOS mRNAs expressions.	

*Himatanthus sucuuba* (Spruce ex Müll. Arg.) Woodson (Apocynaceae)	Bark; latex; leaves	Plumericin (**23**); isoplumericin (**24**)	Wound healing; laxative; hallocinogen; tumours; boils; oedema; arthritis; gastritis; verminosis	No	Yes	-	[109-111]

*Kigelia africana *(Lam.) Benth. (Bignoniaceae)	Bark; Fruit; Flower	Verminoside (**25**)	Psoriasis; eczema; venereal disease	Yes	Yes	Verminoside inhibits iNOS expression and NO release in the LPS induced J774.A1 macrophage cell line.	[112-116]

*Lamiophlomis rotate *(Benth. ex Hook. *f*) (Lamiaceae)	Aerial parts; roots	Loganin (**8**) (and others)	Detumescence; haemostasis; pain alleviation; blood circulation promotion	Yes	Yes	*Lamiophlomis rotata *injection increased phagocytosis by mouse peritoneal macrophages, and decreased the LPS-induced production of IL-1.	[117-118]

*Mentzelia scabra subsp. chilensis *(Gay) Weigend (Loasaceae)	Aerial parts	Mentzeloside (syn. deutzioside) (**26**)	Gastric ulcers; helminth infections	No	Yes	Dose-dependent inhibitory activity on carrageenan induced rat-paw oedema.	[119-120]

*Phillyrea latifolia* L.(Oleaceae)	Aerial parts	Oleuropeoside (**27**); ligustroside (**28**)	Oropharyngeal inflammation; burns; headaches	Yes	No	Inhibition of PGE_2_ release.	[121-123]

*Picrorhiza kurroa *Royle ex Benth. (Scrophulariaceae)	Root	Picroside II (**29**); picroside III (**30**); picroside V (**31**); 6-feruloyl catalpol (**32**); picroside I (**34**); kutkoside (**35**)	Jaundice; indigestion; common fever; acute viral hepatitis and bronchial asthma	Yes	Yes	Stimulation of the cell-mediated and humoral components of the immune system.	[43; 124-129]

*Plantago asiatica *L. (Plantaginaceae)	Seeds	Aucubin (**14**)	Bronchitis; diarrhoea; constipation, wounds	No	Yes	Oral wound healing.	[83; 130-131]

*Rehmannia glutinosa *Steud.(Scrophulariaceae)	Root	Catalpol (**15**)	Contact dermatis and rhinitis; promotes blood circulation; improves kidney function	Yes	Yes	Inhibition of the secretion of both TNF-α and IL-1.	[132-133]
						Neuroprotective by attenuating LPS-induced the expression of iNOS.	

*Russelia equisetiformis *(Schltdl. & Cham.)	Whole plant	10-*O*-cinnamoyl sinuatol (**36**)	Diabetes; leukemia; pain and inflammation	Yes	Yes	Inhibition of pro-inflammatory mediators.	[134-136]

*Scrophularia auriculata* ssp. *pseudoauriculata,* (Scrophulariaceae)	Aerial parts	Scropolioside A (**38**); scrovalentinoside (**39**)	Inflammatory skin diseases	Yes	Yes	*In vivo* scropolioside A reduces both oedema formation and cell infiltration whereas *in vitro* it reduces the proliferation of activated T-lymphocytes.	[39-40]
						Inhibition of the production of PGE_2_, leukotriene B_4_, NO, IL-1β, IL-2, IL-4, TNF-α and interferon-γ.	

*Scrophularia deserti *Delile (Scrophulariaceae),	Aerial parts	Harpagoside (**19**); scropolioside-D2 (**40**); koelzioside (**41**)	Fever; kidney diseases; diabetes mellitus; inflammation of the mouth, lungs, large intestines, bladder and heart	No	Yes	-	[137-139]

*Scrophularia frutescens* L. (Scrophulariaceae)	Aerial parts	Harpagoside (**19**)	Joint and lower back pain and inflammation	Yes	Yes	Hydrolysed products of harpagoside with glucosidase treatment showed a significant inhibitory effect on COX-2 activity.	[44; 140-141]

*Scrophularia scorodonia* L. (Scrophulariaceae)	Aerial parts	Aucubin (**14**);harpagoside (**19**); harpagide (**20**); 8-acetylharpagide (**42**); scorodioside (**43**); scropolioside B (**44**); bartsioside (**45**)	Inflammatory diseases	Yes	No	Inhibition of TXB_2_, PGE_2_ and LTC_4_ release.	[142-143]

*Sideritis perfoliata *L. subsp. *perfoliata *(Lamiaceae)	-	Ajugoside (**46**)	Rheumatism; ulcers; digestive disorders	Yes	No	Lipoxygenase inhibition.	[144]

*Stachytarpheta cayennensis* (L.C. Rich) Vahl (Verbenaceae)	Flower; leaves; roots	Ipolamiide (**47**)	Pain; inflammation; fever; liver and gastric disturbances	Yes	Yes	Inhibition of leukocyte accumulation and influx.	[56; 145-148]
						Inhibitory effect on histamine and bradykinin.	

*Syringa *species(Oleaceae)	Leaves	Iridoid glycoside-rich fraction; Syringopicroside (**48**)	Acute enteritis; icteric hepatitis; acute mastitis; bacillary dysentery; upper respiratory tract infections	Yes	Yes	Reduction of the activity of myeloperoxidase, depression of malondialdehyde and NO levels and inhibition of the protein and mRNA expressions of NFK-B and TNF-α and IL-6.	[149-151]

*Verbascum lasianthum* Boiss. (Scrophulariaceae)	Aerial parts	Aucubin (**14**); catalpol (**15**); geniposidic acid (**17**); ajugol (**49**)	Respiratory disorders; urinary tract infections; earache; inflammatory skin disorders	No	Yes	-	[155]

*Verbascum mucronatum* Lam (Scrophulariaceae)	-	Catalpol (**15**); ajugol (**49**); lasianthoside (**50**); picroside IV (**51**)	Respiratory disorders; urinary tract infections; earache and inflammatory skin disorders	-	Yes	-	[152; 156]

*Verbascum pterocalycinum* var. *mutense *Hub.-Mor. (Scrophulariaceae)	Flowers	Ajugol (**49**); picroside IV (**51**)	Respiratory disorders; urinary tract infections; earache, inflammatory skin disorders	No	Yes	-	[157-158]

*Verbena officinalis* L. (Verbenaceae)	Whole plant	Verbenalin (**52**)	Detoxing agent; diuretic; expectorant and anti-rheumatic	No	Yes	Topically and orally administered extracts showed anti-inflammatory activity in the TPA-induced ear inflammation model and in carrageenan-induced rat-paw oedema.	[159-163]

*Veronica anagallis-aquatic* L. (Plantaginaceae)	Aerial parts	Catalposide (**6**);veronicoside (**53**); verproside (**54**)	Influenza; pain; haemoptysis; laryngopharyngitis and hernia	-	Yes	Catalposide significantly inhibited the production of NO in LPS-stimulated RAW 264.7 macrophages in a dose-dependent manner. RT-PCR and Western blot analyses demonstrates that catalposide also suppressed the expression of the iNOS gene and protein and inhibited the activation of LPS-induced NF-κB.	[43; 66; 163]

*Vitex peduncularis *Wall. ex Schauer (Verbenaceae)	Root bark or young stem bark	Pedunculariside (**55**); agnuside (**56**)	Malaria type fever, especially black water fever	Yes	No	Selective inhibition of COX-2.	[164-165]
